# Review of Applications of Microneedling in Melasma

**DOI:** 10.1111/jocd.16707

**Published:** 2024-12-27

**Authors:** Wenwen Chen, Xingling Jian, Bo Yu

**Affiliations:** ^1^ Department of Clinical Medicine Shandong Second Medical University Weifang China; ^2^ Department of Dermatology Peking University Shenzhen Hospital Shenzhen China

**Keywords:** application, melasma, microneedle, review

## Abstract

**Background:**

Melasma, a common skin pigmentation disease, can negatively impact patients' mental health, social interactions, and physical appearance. Although we now have several treatments accessible, such as medicines, chemical peels, and phototherapy, which can help ease symptoms to some extent, the requirement for a long‐term effective and safe treatment for patients is far from met. In the face of this problem, microneedling, as an innovative treatment, provides a new avenue for treating melasma. Although microneedling has been extensively investigated for treating other skin issues such as inflammation, scarring, and photoaging, research into its use in melasma treatment is still in its early stages.

**Objective:**

This study aimed to gather and assess clinical information on microneedling's effectiveness in treating melasma, covering research gaps and serving as a beneficial reference for clinical therapy.

**Methods:**

We searched PubMed, Cochrane, Scopus, Embase, and Web of Science databases for articles with the keywords “microneedling,” “percutaneous collagen induction”, and “melasma.” Following a thorough assessment, we selected 64 clinical studies that matched the requirements for in‐depth analysis.

**Results:**

After thoroughly reviewing these data, we concluded that microneedling has tremendous potential for treating melasma. Microneedling can significantly improve treatment outcomes, especially when paired with additional therapies such as topical medicines or phototherapy.

**Conclusion:**

Overall, the evidence reported in this study demonstrates that microneedling is an essential advancement in melasma treatment. Not only can it improve the efficacy of topical drugs and other treatment modalities, but it also has an excellent safety and tolerability profile, making it desirable to patients and clinicians. While the current findings are encouraging, more study is needed to refine treatment protocols, investigate the long‐term consequences of microneedling, and establish it as the standard of care for melasma treatment. We anticipate that microneedling will play an increasingly important role in the future of melasma treatment, providing our patients with more hope and a broader choice of treatment alternatives.

## 1 Introduction

Melasma, a skin pigmentation condition that affects people's physical appearance, can also cause psychological problems, including anxiety and sadness. Consequently, melasma management and treatment are significant challenges for the medical community and the general public. Among Asian women of childbearing age, melasma prevalence is 30% [1]. Melasma, which has a complex etiopathogenesis and is multifactorial, has a wide range of clinical symptoms and histological features [2]. Although there are many melasma treatments, including chemical peel therapy, phototherapy, systemic medication therapy, and cosmetic therapy, their long‐term safety and efficacy are yet to be tested. Researchers are investigating new ways of overcoming current treatment limitations, such as via the development of targeted topical components, combined phototherapy, and combining microneedle therapy and pharmaceutical medications.

Microneedling therapy, also known as percutaneous collagen induction therapy, is a new treatment that encourages the growth of collagen tissue in the skin by physical or mechanical methods. This therapy uses a device with many fine needles to create micropunctures on the skin, promoting collagen regeneration. This approach preserves the epidermis' integrity and considerably improves the percutaneous absorption of therapeutic drugs, providing good clinical results in the treatment [3]. Microneedle therapy, initially known as “incision,” was developed by Orentreich et al. [4] for scar and wrinkle healing. However, it is inappropriate for extensive face rejuvenation or treating minor body areas. Dr. Desmond Fernands pioneered a unique procedure, whereby the skin is continuously pierced with a drum‐shaped device equipped with many tiny needles, which preserves the epidermis while promoting collagen renewal [5]. This breakthrough accelerated microneedle therapy development, leading to various products, including motorized, radiofrequency, and nano microneedles [6]. Moreover, because of its low cost and minimal invasiveness, microneedle therapy has the potential for use in dermatological and cosmetic treatments.

Although traditional melasma treatments like hydroquinone are considered safe and effective, their therapeutic potential is limited by many factors, such as sluggish onset and poor patient compliance. Their long‐term use may also have adverse side effects, including dryness, peeling, and uneven skin pigmentation, highlighting the need for alternative treatments [7]. Here, based on the most recent research findings, we assess the full use of microneedle therapy on patients with melasma and offer an overview of various microneedling types, their potential therapeutic mechanisms against melasma, and their real‐world clinical uses.

## 2 Methods

Covering the period from database creation to May 2024, literature searches were performed on PubMed, Cochrane, Scopus, Embase, and Web of Science databases for papers on microneedling use for melasma treatment. Additional relevant data were obtained from the reference lists of the included publications. The search criteria included the MeSH phrases (microneedle* or microneedling or “skin needling” or percutaneous collagen induction or micro needling or dermapen or dermaroller or “dermal needling” or needling or “needling, skin” or collagen induction, percutaneous) AND (melasma* or chloasma* or melanosis or melanism* or freckle* or melanoses), adjusted accordingly in each database. Only articles published in English were included. Included clinical investigations involved the use of microneedles alone, microneedle use in combination with medication (oral, topical, andinjectable) or as a multimodal treatment component, and comprehensive accessible data involving human participants. This review is based on a large corpus of literature and for a thorough analysis, 64 significant microneedle and melasma datapoints were extracted.

## 3 Results

### 3.1 Study Selection

A thorough literature search involving keywords and free‐text queries yielded 629 results. The screening procedure, which strictly adhered to the established inclusion and exclusion criteria, identified 80 articles for initial evaluation. After a thorough review, eight studies that used facial acupuncture or acupuncture to specifically treat melasma, seven that used mesodermal therapy to manage melasma, and one that lacked complete text, were excluded. Finally, 64 publications were selected for comprehensive review.(See Tables 1, 2, 3 for details).

**TABLE 1:** Single microneedle treatment.

Reference	Microneedle type	Number of subjects	No of sessions (interval)	Treatments	Results	Side effects
Cassiano et al. [22]	Dr. Roller, 1.5 mm	20 females	before/after 7 days	MN for full face	MN significantly reduced melanin density, prolapsed melanocytes, and basement membrane damage in tissues	Not mentioned
Taher, El‐Sayed and Bessar [23]	Dermapen	17 females	5 (2 weeks)	Right side: MN Left side: Placebo	Compared with placebo, MN alone had a significant whitening effect with satisfactory results	Not mentioned
Park et al. [92]	RF,1.0–1.5 mm	25 patients	5 (2 weeks)	RF for full face	RF could be a treatment option for facial pigmentary disorders	Mild erythema, edema, pain
Wu et al. [25]	MFRS, 1.5 mm	Nine patients	2 (3 months)	MFRS for full face	MRFS therapy did not lead to hyperpigmentation or aggravated melasma in patients with or without melasma	Erythema, edema, or purpura
Markowitz [30]	RF with Profondmartrix Systham	Two patients	3 (6 weeks)	RF for full face	The device can significantly reduce the density of blood vessels in the skin of melasma patients	No adverse effects were observed
Han et al. [29]	RF, 0.3, 0.8 mm	11 females	12 (2 weeks)	After 4 weeks of oral TA + triple cream, randomized to left or right side for radiofrequency treatment	The continuous RF is beneficial in maintaining the conventional therapy of melasma	Mild erythema and pain

Abbreviations: MFRS, microneedle fractional radiofrequency system; MN, microneedling; RF, radiofrequency; TA, tranexamic acid.

### 3.2 Study Characteristics

This review provides a complete overview of the included studies, including treatment cycles, the number of treatment sessions, device type, and treatment adverse events. The 64 studies included in this review involved > 1000 patients with melasma and of these, > 90% were female, indicating that melasma is more prevalent among women. The microneedle devices used in the included studies were mainly Demaroller (the most popular microneedle instrument worldwide), Dermapen, or their variants. Needle lengths ranged from 0.25 to 2 mm, indicating that although microneedle device types for melasma treatment are relatively constant, needle lengths vary broadly, allowing for personalized selection based on individual patient needs. The studies' treatment cycles varied widely, with the number of treatment sessions and the time between treatments ranging from 1–12 to 0–4 weeks, respectively, highlighting the importance of individualized treatment based on unique patient characteristics and reactions. The clinical studies that used microneedling for melasma reported erythema and discomfort as the most common side effects. However, the prevalence of serious adverse responses was low, implying that although some side effects may occur during treatment, they are often tolerable to patients. The included studies offer crucial clinical insights that provide the foundation for future melasma treatment research.

## 4 Discussion

### 4.1 Microneedling Tools

The microneedling market has markedly grown because of advancements in medical and cosmetic technology, giving rise to a wide variety of microneedling products that fall into the following main categories: dermaroller, dermapen, and combined‐type microneedling devices (radiofrequency microneedling, LED microneedling, and the microneedle drug delivery system) [8, 9, 10, 11, 12]. The characteristics of microneedle products vary from company in features, including material composition, needle diameter, length, and structural design.

We discovered that more researchers preferred the dermaroller to alternative microneedling products due to the following factors:

1. Easy to use: The dermaroller is a manually operated microneedling device that allows the doctor or user to adjust the angle, force, and depth based on the individual's skin condition and comfort level, as well as the various treatment areas, particularly in sensitive areas like the eyes, nose, and lips. This personalized approach is ideal for individuals seeking a tailored treatment plan. Dermapen and combined‐type microneedling devices, on the other hand, necessitate more complex operation and setup and may require the assistance of specialized professionals. Furthermore, the dermaroller's handle is ergonomically built for a secure grip, reducing hand fatigue and increasing user comfort, this design makes it more flexible and easier to use during therapy.
2. Flexibility in treatment: Dermaroller offers microneedle lengths ranging from 0.1 to 3.0 mm, including CIT‐8TM (0.5 mm), MF8TM (1.5 mm), MF‐4 (1.5 mm), and Beauty Mouse (0.2 mm) for home care [6, 8, 13]. This design enables the dermaroller to be tailored to various skin thicknesses and treatment requirements, whether for professional medical aesthetics or daily home care.
3. Lower cost‐effectiveness: Dermaroller is less expensive to maintain than dermapen, which not requires frequent needle changes or the purchase of extra accessories. At the same time, compared to high‐end technologies like RF microneedling and LED microneedling, dermaroller offers patients a more inexpensive option while still providing effective treatment.

### 4.2 Microneedling Mechanisms of Action in Melasma Treatment

Although the mechanisms by which microneedling works in melasma treatment remain unclear, it is widely accepted that its main therapeutic benefits come from enhanced transdermal absorption of drugs or bioactive substances and wound healing response stimulation [14, 15]. The effectiveness of these mechanisms depends on microneedle penetration depth into skin tissue, which in turn, depends on several parameters, including needle length, applied force, and subcutaneous fat levels in the targeted region. It is widely believed that needle tips pierce at a depth of 50%–70% of their total length [16].

Like a brick wall, intact skin stratum corneum acts as a protective barrier and is critical for maintaining vital physiological processes like reducing water and electrolyte loss, resisting mechanical stress, and exerting anti‐inflammatory effects [17]. Being hydrophobic, it naturally restricts the absorption of most pharmacologic agents and active substances, limiting transdermal absorption rates. Consequently, only small medication doses reach target tissues to provide therapeutic effects, which frequently results in unsatisfactory clinical outcomes. Because microneedles with a length of ≤ 1 mm penetrate and temporarily break down the epidermal barrier, they increase the transdermal absorption of medicines and bioactive chemicals [18]. Although they may also affect the epidermis–dermis junction, they cause less bleeding because the superficial dermal layer is only partially affected. This mechanism may boost basal cell activity, rejuvenate aging keratinocytes, minimize stratum corneum accumulation, and aid melanosome evacuation. By piercing the epidermis and reaching the dermis, microneedles with a length of > 1 mm can trigger a sequence of post‐traumatic healing processes, which stimulates the release of various factors, including platelets, transforming growth factor α and β, fibroblast growth factor, interleukin 1, and tumor necrosis factor [18, 19, 20]. These factors promote neovascularization, nutrient and oxygen supply for cellular metabolism, cell migration and proliferation, and collagen and elastin formation. Fibroblast migration and proliferation stimulate extracellular matrix synthesis, including the production of mucopolysaccharides and the expression of collagen and elastin, driving skin tissue remodeling. Furthermore, microneedling can reduce scar formation by increasing transforming growth factor β3 expression, which is critical for enhanced skin pigmentation and overall skin health maintenance [21].

In a thorough review of 64 clinical studies, we discovered that researchers preferred using 1.5 mm microneedles for melasma treatment and that patients' symptoms improved significantly within 8–12 weeks. This phenomenon could be attributed to the fact that 1.5 mm microneedles can precisely enter the skin to the correct depth, efficiently triggering the dermal repair mechanism without causing excessive skin damage, which is critical for best therapy results.

### 4.3 Microneedle Therapy in Melasma

#### 4.3.1 Single Microneedle Treatment

In melasma treatment, microneedle therapy has several advantages, especially in skin tissue structure modification, melanin deposition reduction, and boosting melanin particle elimination, which can significantly improve melasma symptoms and increase the quality of life [22, 23]. Cassiano et al. [22] administered microneedle therapy to 20 female patients with melasma and through histopathological examination before and after treatment, found that microneedle treatment markedly reduced melanin density, thickened the epidermis, and reduced basement membrane damage, highlighting its efficacy in melasma treatment.

However, microneedle therapy is invasive, which may harm skin barrier function, increasing the risk of infection and inflammation [24]. To address this challenge, Wu et al. [25] treated nine patients with melasma using a microneedle fractional radiofrequency system and found that after treatment, patient face transdermal water loss (TEWL) increased temporarily before returning to baseline levels after a week. This finding implies that in the early stages, the microneedle fractional radiofrequency system may impair epidermal barrier function, causing water loss and increased TEWL. However, the return of TEWL values to baseline levels after a week with subsiding cell swelling and epidermal function recovery indicates that skin sensitivity was not altered in the long run. Therefore, although microneedle therapy can damage the epidermal barrier in a short period, it does not easily cause infection.

Therapeutic impact maintenance and limiting recurrence is critical for melasma treatment and traditional therapeutic methods do not address this issue effectively. However, new research findings indicate that dermal regeneration can be enhanced using targeted therapies, such as radiofrequency, pulsed dye lasers, and pulsed solid light, maintaining therapeutic impact for longer [26, 27, 28]. For example, Han et al. [29] treated 11 female patients with melasma for 2 months using a routine treatment that included oral tranexamic acid and a topical triple cream followed by microneedle radiofrequency for maintenance and found that even after conventional therapy discontinuation, the radiofrequency study arm remained better at 6 months, whereas in the untreated arm, 63.6% of the participants returned to baseline levels. This indicates that radiofrequency can provide long‐term therapeutic effects while lowering recurrence likelihood. Furthermore, using radiofrequency therapy on two patients with melasma, Markowitz et al. [30] found that skin blood vessel density was markedly reduced following treatment, probably because of radiofrequency use with the Profondmartrix System, which can target three tissue layers in a single operation and effectively releases heat, destroying vascular tissue and minimizing the epidermal cell damage caused by multiple insertions and pulse stacking.

Finally, microneedle monotherapy is effective in melasma treatment and can be used as maintenance therapy for other treatment modalities (See Table 1 for details). Future studies should improve patient satisfaction and outcomes by optimizing treatment schedules.

#### 4.3.2 Microneedle Drug Delivery

Melasma is a complex pigmentary disorder influenced by melanosome synthesis, transportation, and metabolism, UV damage, basement membrane disruption, inflammatory responses, improper lipid metabolism, and other factors [31, 32]. Considering the complexity of melasma development, microneedles alone might not fully meet melasma therapeutic requirements and for maximum therapeutic benefit, it should be combined with additional functional components or medications, these can be used alone or in combination.

Various medications and active compounds have demonstrated therapeutic effectiveness in treating melasma through their distinct mechanisms. Numerous studies have investigated the combination of microneedle therapy with hydroquinone (HQ), tranexamic acid (TA), vitamin C, ascorbic acid (AA), and platelet‐rich plasma (PRP), showing notable success with this approach. However, the interaction of microneedle therapy with other treatment agents has not been extensively studied. This gap has piqued our interest, as utilizing microneedles alongside multiple components could present new methods to enhance treatment effectiveness. After thoroughly reviewing 64 clinical studies, as detailed in Table 2, we identified the primary categories of ingredients currently being used in conjunction with microneedling, which include: HQ; nonhydroquinone drugs or ingredients (TA, retinoic acid [ATRA] [33, 34], methimazole [MMI] [35], glutathione [36]); cosmeceuticals and botanicals (AA, glabridin [37], rucinol [4‐n‐butylresorcinol] [38]);biological therapy (PRP, stem cell exosomes, polynucleotide [PN], hyaluronic acid [HA]).

**TABLE 2:** Microneedle drug delivery.

Reference	Microneedle type	Number of subjects	No of sessions (interval)	Treatments	Results	Side effect
Lima [39]	Dr. Roller, 1.5 mm	18 females Four males	2 (4 weeks)	MN + triple decolorizing formula (0.05% ATRA + 4% HQ + 1% FLU)	MN can treat stubborn melasma	Erythema and discrete punctuated bleeding
Lima et al. [40]	Dr. Roller, 1.5 mm	Six females	2 (4 weeks)	MN + triple decolorizing formula	All patients showed improvement in melasma	Not mentioned
Ramírez‐ Oliveros et al. [41]	Not mentioned, 1.5 mm	One female	1	MN + 4% HQ sterile serum drug + Kligman's formula	Significant improvements were observed in the clinical outcomes and quality of life	Not mentioned
Bosamiya and Jain [42]	Dermaroller, 1.5 mm	32 females Eight males	3 (4 weeks)	Group I: MN + depigmentating cream; Group II: Depigmentating cream (0.05% ATRA + 2% HQ + 0.01% FLU)	MN provides faster results than topical creams alone	Mild discomfort and edema: group I, lasting for 2 ~ 3 days
Budamakuntla et al. [43]	Dermaroller, 1.5 mm	54 females Six males	3 (4 weeks)	Group I: MI + 4 mg/mL TA; Group II: MN + 4 mg/mL TA	MASI was elevated in both groups, 35.72% and 44.41%, respectively	Mild erythema, tolerable pain
Xu et al. [44]	Not mentioned, 0.25 mm	28 females	12 (1 week)	Patients received functional microarray of MN + 0.5% TA on one side of their face and 0.5% TA on the other	This device improves the effectiveness of TA in treating melasma	Dermatographism: 1 patient
Saraiva et al. [45]	Eletroderme, 2 mm	15 females	8 (15 days)	4 mg/mL TA + Eletroderme (without RF)	MASI significantly reduced from 21.33% to 11.19%	Pain tolerance
Saleh et al. [46]	Dermapen, 1.5 mm	42 females	4 (2 weeks)	Group I: MN + 10% TA; Group II: MN	TA + MN is superior to MN alone	Mild erythema, burning sensation, discomfort tolerated in most patients
Wali and Parwani [47]	Dermaroller, 2.5 mm	12 females Eight male	3 (4 weeks)	Group I: Oral TA + topical triple cream; Group II: MN + 4 mg/mL TA	Triple cream is safe and effective, MN or TA can assist in its treatment	HP: 40.0% (I) + 30% (II)
Kaur et al. [48]	Dermaroller, 1 mm	32 females Eight males	4 (2 weeks)	Patients received MN + 0.5% TA on one side of the face and MN + distilled water on the other side of the face	MN + TA and MN alone are effective in treating melasma	HP: 5% patients; Dryness: 55% patients
Cassiano et al. [49]	Not mentioned, 1.5 mm	64 females	2 (4 weeks)	Group I: MN + Oral placebo; Group II: Oral TA Group III: MN + Oral TA; Group IV: Oral placebo	TA and MN in different pathways that lead to improvements in melasma	Oral TA: 3% persistent headache MN: 9.3% herpes simplex
Shamsi Meymandi et al. [50]	Dr. Roller, 1.5 mm	60 females	4 (4 weeks)	Group I: MN + 4% TA; Group II: Topical 4% HQ	MN + TA did not differ from 4% HQ in the treatment of melasma.	MN + TA had more erythema but less HP than HQ.
Ebrahim et al. [51]	Dermapen, 1.5 mm	56 females	6 (2 weeks)	Right side: MI + 4 mg/mL TA; Left side: MN + 4 mg/mL TA	MI + TA and MN+ TA could be safe and effective in melasma treatment.	Mild erythema, edema, pain, mild irritation
Farag, AbdElMaksoud and Ismail [52]	Dermaroller, 1 mm	15 females	8 (1 week)	Right side: MN + mesolighten (3% KA + 0.01% TA + 4% Azo + 1 g LAA + water for injection); Left side: MN + 4 mg/mL TA	MN+ TA for melasma superior to MN + mesolightening	Pain, HP, Persistent erythema, Allergy to anesthesia
Xing et al. [53]	Dermapen, 1 mm	60 (not mentioned)	12 (1 week)	Group I: 1.8% liposomal TA; Group II: MN + 5% TA solution Group III: 2% HQ	Better treatment outcomes in Group III compared to Group I and III	Erythema aggravation and HP in Group III
Hassan et al. [54]	Dermapen, 0.25–2 mm	57 females 9 males	3 (4 weeks)	MN + 4 mg/mL TA	Microneedling combined with TA is highly effective in most patients	Mild discomfort, burning sensation and erythema
Zaky et al. [55]	Dermapen, 1.5 mm	50 females	4 (1 week)	Group I: MN + 4% TA; Group II: 4% HQ	4% HQ and MN+ TA are both effective and safe in melasma	HP: 4% (I),12% (II)
Kuster Kaminski Arida et al. [56]	Dermaroller, 1.5 mm	20 females	3 (4 weeks)	Patients received MN + 50 mg/mL TA on one side of their face, MN + 0.9% saline on the other	MN + TA provided no additional benefit in melasma	Not mentioned
Susmitha et al. [57]	Dermaroller, 1.5 mm	43 females 2 males	12 (1 week)	Group I: modified kligman formula (2% HQ + 0.025% ARTA + 0.1% MOM); Group II: MN +4 mg/mL TA; Group III: ID +4–8 mg/mL TA	Both have comparable efficacy and can be used as primary or maintenance therapy	Not mentioned
Cassiano et al. [59]	Dr. Roller, 1.5 mm	64 females	8 (2 weeks)	Group I: Oral placebo, Group II: MN + Oral placebo, Group III: Oral TA, Group IV: MN + Oral TA + Triple combination cream nightly	These treatments improve epidermal and dermal tissue	Not mentioned
Batra et al. [58]	Dermaroller, 1 mm	31 females 9 males	6 (2 weeks)	Group I: Oral TA; Group II: MN + 4 mg/mL TA	Oral and transdermal TA are equally effective	Epigastric discomfort: 10% group I Pain: 35% group II
Poostiyan et al. [60]	Dr. Roller, 1.5–2 mm	27 females	3 (4 weeks)	Group I: Right side: MN + 100 mg/mL TA, Left side: MI + TA; Group II: Right side: MI + 100 mg/mL TA, Left side: MN + TA	Comparable results of MI+ TA and MN+ TA in the treatment of facial melasma	MI showed higher erythema, desquamation, and edema than MN, and PIH was only discovered in MI
Yu et al. [61]	Polylactic acid MN, 0.4 mm	11 females 1 male	Twice daily for 8 weeks	Patient received polylactic acid MN + 2.9% TA serum on one side of the face and the other side received 2.9% TA serum	Improvement of melasma with PLA MN + TA serum is superior to that with TA serum only	Flaking: 8.3%, improves with increased moisturization
Zheng et al. [62]	Dr. Roller,0.5 mm	7 females	3 (4 weeks)	MN + 5% TA	MN + TA improves melasma	Not mentioned
Kusumawardani et al. [33]	0.5–1 mm	3 females	Not mentioned	Liposomal serum containing combination of azelaic acid, 4‐n‐butylresorcinol and retinol + microneedling	MASI, MSS and Melas‐QoL improved in all patients with the scores of 33.3%–85%	Not mentioned
Bergmann et al. [34]	Dr. Roller, 1 mm	42 females	4 (15 days)	Group I: MN + 5% ATRA; Group II: 5% ARTA stripping solution	Both improved melasma, but MN was ineffective against non‐enzymatic defenses	Not mentioned
Farag et al. [35]	Dermapen, 0.25 ~ 0.5 mm	30 females	12 (1 week)	Right side: MN + 5% MII; Left side: MN + Placebo	MN + MII can significantly improve melasma	Erythema: 3.3%; HP:3.3%
Mohamed, Beshay and Assaf [36]	Dermapen, 0.5–2 mm	29 females	6 (2 weeks)	Right side: MN + Glutathione; Left side: MN	MN + glutathione improve melasma better than MN only	Slight erythema, edema, and dryness
Yan et al. [63]	Dr. Roller,0.5 mm	51 females	5 (2 weeks)	Group I: MN + 20 mg/mL AA + Human Collagen Repair Dressing + HQ; Group II: Human Collagen Repair Dressing + HQ	MN in combination with AA, humanoid collagen and HQ improves melasma efficacy	Transient erythema: 22.2% (I) Mild itchiness: 12.5% (II)
Ismail et al. [64]	Dermaroller, 1.5 mm	30 females	6 (2 weeks)	MN + Pure L‐Ascorbic acid 20%	MN + AA is safe and effective in treating epidermal melasma	HP: 16.7%
Farshi and Mansouri [65]	Dermapen, 1.5 mm	19 females 1 male	4 (4 weeks)	Patients received MN + mesoestetic depigmentatisolution on one side, and Meso + mesoestetic depigmentati solution on the other	Both MN and Meso decreased the melanin content in epidermal melasma lesions	Erythema: 20%; Skin dryness: 15% Itching: 20%; Burning sensation: 25%
Menon et al. [66]	Dermaroller, 1.5 mm	30 females	4 (2 weeks)	Right side: MN + 20% AA; Left side: MN + 10% TA	MN + TA improves melasma better than MN + AA	Itching and burning sensation: 33.3%
Tahoun Mostafa and Amer [67]	Dermapen, 1.5 mm	30 females	5 (2 weeks)	Right side: MN + 100 mg/mL TA; Left side: MN + 20% AA	Both improve melasma, MN + TA is better for vascular and epidermal pigmentation	Itching, Erythema, HP
El et al. [68]	Dermapen,1.5 mm	20 females	6 (2 weeks)	Right side: MN + 100 mg/mL TA; Left side: MN + 30% AA	Both can improve melasma, TA works better on blood vessels	Not mentioned
Raza et al. [69]	Dermapen,0.5 mm	19 females 11 males	3 (2 weeks)	Right side: MN + 100 mg/mL TA; Left side: MN + 20% AA	Both are safe and effective in treating melasma	No significant side effects
Gul et al. [70]	Dermaroller	60 females	6 (2 weeks)	MN + 20% AA	MN + AA is safe and effective in treating melasma	Pain, transient erythema
Yan et al. [37]	DMN,0.23 mm	3 patients	28 (2 days)	DMN patch was placed on the area of pigmentation beneath the right eye	DMNs were found safe in clinical trials and selectively lightened chloasma and age spots	Not mentioned
Fabbrocini et al. [38]	CIT 8, 0.5 mm	20 females	2 (4 weeks)	Right side: MN + decolourizing serum; Left side: Decolourizing serum((rucinol and sophora‐alpha)	MN+ decolourizing serum is better than depigmenting serum alone	Mild erythema
Honfny et al. [71]	Dermapen, 2 mm	20 females	3 (4 weeks)	Right side: MN + PRP; Left side: Meso + PRP	MN + PRP was increased the expression of TGF‐β protein	Not mentioned
Honfny et al. [72]	Dermapen,2 mm	19 females 4 males	3 (4 weeks)	Right side: MN + PRP; Left side: Meso + PRP	Both improve melasma, but patient satisfaction is higher with MN + PRP	Pain, erythema, and edema
Boparai, Bhale and Brar [73]	Dermaroller, 1.5 mm	18 females 12 males	3 (3 weeks)	MN + PRP	PRP is safe and effective in treating melasma	Mild erythema: 80% patients
Gharib, Mostafa and Ghonemy [74]	Not mentioned,1.5 mm	26 females	Four patients	Group I: MN + 4 mg/mL TA; Group II: MN + PRP	MN + PRP improves melasma better than MN + TA	TA exhibited higher erythema than PRP but less pain and HP
Panda et al. [75]	Dermaroller,1 mm	31 females 20 males	3 (4 weeks)	Group I: MN + PRP; Group II: MN	MN + PRP is safe and effective in treating melasma	Mild erythema, edema, and pain
Tekam and Belgaumkar [76]	Dermapen, 0.5 ~ 2 mm	17 females 13 males	4 (3 weeks)	Right side: MN + PRP + 4% HQ; Left side: MN + Saline+4% HQ	MN + PRP + HQ is superior to MN + HQ for melasma improvement	Mild erythema
Ragaie et al. [69]	Dermapen,1 mm	10 females	6 (2 weeks)	Right side: MN + 20% AA; Left side: MN + PRP	MN + AA is better than MN + PRP in improving mixed melasma	Transient erythema and burning sensation: 30%
Wang et al. [77]	Dermaroller,1 mm	60 females	4 (4 weeks)	Group I:NAFL + normal saline; Group II: MN + hUCMSC‐Exos Group III: NAFL + hUCMSC‐Exos; Group IV: PBASM + hUCMSC‐Exos	hUCMSC‐Exos is safe and effective. MN, NAFL, and PBASM have similar pro‐osmotic effects	Erythema: 40% (I), 13.3% (II), 13.3% (III); Pain and irritation: 6.7% (II)
Gulfan et al. [78]	RF,0.8 mm, 1.5 mm	30 females	3 (2 weeks)	Patient received RF + PN on one side of the face and the other side received RF	Both are safe and effective in improving melasma	Mild erythema and pain, relapsing
Avcil et al. [79]	HA‐MA, 0.35 mm	10 patients	12 weeks (1 time per day)	HA‐MA in melasma areas	HA‐MA improves melasma and enhances skin brightness	No associated dermatology‐related skin changes were caused

Abbreviations: AA, vitamin C; ARTA, retinoic acid; DMN, dissolving microneedles; FLU, fluocinolone acetonide; FMN, functional microarray of microneedles; HA, hyaluronic acid; HA‐MA, hyaluronic acid‐based microneedles; HP, hyperpigmentation; HQ, hydroquinone; hUCMSC‐Exos, human umbilical cord mesenchymal stem cell‐derived exosomes; ID, intradermal injection; Meso, mesotherapy; MI, microinjection; MMI, methylimidazole; MN, microneedling; NAFL, nonablative fractional laser; PBASM, Peninsula Blue Aurora Shumin Master; PN, polynucleotide; PRP, platelet‐rich plasma (PRP); TA, tranexamic acid.

HQ has long been considered the gold standard for treating melasma, with doses ranging from 2% to 4% [39, 40, 41, 42]. It is commonly mixed with retinoids and steroids to create the well‐known triple cream. Bosamiya et al. [42] found that when compared with triple cream alone, combining microneedles with triple cream resulted in a higher Melasma Area and Severity Index (MASI). This novel combined therapy not only builds on hydroquinone's documented efficacy but also uses microneedling's increased permeability, providing patients with a more effective and less irritating therapeutic choice.

TA is emerging as a new star in the treatment of melasma due to its various modes of action and excellent safety record [43, 44, 45, 46, 47, 48, 49, 50, 51, 52, 53, 54, 55, 56, 57, 58, 59, 60, 61, 62]. In clinical practice, topical TA concentrations range from 0.4% to 10%, with varied therapeutic benefits. Higher doses of TA may theoretically result in greater efficacy due to better tyrosinase inhibition. However, they may also be associated with an increased risk of side effects, as demonstrated by research by Saleh et al. and Poostiyan et al. [46, 60]. It is worth noting that more and more studies are favoring the use of TA concentrations of 4 mg/mL (0.4%), which could be because when combined with microneedling, satisfactory therapeutic results can be achieved even with lower TA concentrations, as supported by the study of Budamakuntla et al. [43]. Furthermore, complex formulations containing TA have emerged as a new trend in the treatment of melasma due to their combination with other active ingredients such as vitamin C, glutathione, and tretinoin, which not only improve treatment efficacy through synergistic effects but may also reduce the risk of side effects. However, Farag et al. [52] found that microneedling combined with a composite formulation of 0.01% TA was no more effective than microneedling treatment with 0.4% TA alone. This discovery challenges our traditional beliefs of combination formulations. It implies that while seeking therapeutic efficacy, we should also prioritize the simplicity and safety of formulations to ensure patients receive both effective and gentle therapies.

AA has garnered popularity in skincare due to its versatility, with concentrations varying from 3.75% to 30% [37, 63, 64, 65, 66, 67, 68, 69, 70]. We find that researchers have particularly endorsed the 20% concentration. Ismail ESA et al. [64] has demonstrated that the combination of 20% AA with micro needling in melasma treatment significantly enhances skin quality and diminishes the size and color intensity of melasma lesions, affirming the safety and efficacy of this high AA concentration. Menon et al.'s [66] research revealed that microneedling with 20% AA or 10% TA was as effective as melasma, yet 20% AA presented fewer side effects, underscoring the superior safety and tolerability of the higher AA concentration. In a groundbreaking development, dissolvable microneedles (DMN), which incorporate many components, including AA, as Yan et al. [37] devised, present patients with a straightforward and potent therapeutic alternative. This cutting‐edge technology enables medications to target the deeper layers of the skin more accurately, thereby augmenting the efficacy of therapy and minimizing potential harm to healthy skin. This treatment paradigm not only deepens our comprehension of AA's role in microneedling but also expands the array of treatment options available to individuals afflicted with melasma.

The emergence of biotherapeutics, such as PRP, stem cell therapies, PN, and HA, has changed the treatment landscape for melasma [71, 72, 73, 74, 75, 76, 77, 78, 79]. PRP therapy has proven to be a game‐changer, effectively reducing hyperpigmentation and enhancing patients' quality of life. Honfny ERM et al. [71] have discovered that the synergy of microneedling with PRP therapy significantly increases TGF‐β protein expression, thereby markedly improving melasma symptoms. This breakthrough sets the stage for further exploration, where subsequent studies have shown that microneedle injections are just as effective as traditional intradermal PRP injections, highlighting that the delivery method does not dictate the success of the treatment [72]. Crucially, the integration of microneedles with PRP has been shown to significantly minimize patient discomfort, pain, bleeding, and infection risk, as opposed to conventional injections. This advancement has bolstered the therapy's safety and increased patient acceptance. Panda et al. [75] have added to this body of evidence, noting a substantial rise in patient satisfaction and treatment efficacy when microneedles are used with PRP. These findings collectively endorse the efficacy of the combined treatment approach, painting a promising future for this method in melasma treatment. In addition to PRP, the union of microneedling with stem cell therapy has unveiled impressive therapeutic outcomes. Wang et al. [77] demonstrated that the marriage of human umbilical cord mesenchymal stem cell exosomes (hUCMSC‐Exos) and microneedling is both safe and efficacious in combating melasma. Moreover, the study underscored that microneedling, when combined with nonablative fractional laser (NAFL) and plasma devices (PBASM), can significantly enhance the transdermal penetration of exosomes, unveiling a cutting‐edge strategy for melasma therapy. These discoveries have introduced innovative concepts for melasma treatment and underscore the pivotal role of biotherapeutics in enhancing treatment efficacy and patient comfort.

In conclusion, the combination of microneedling and beneficial compounds or medications offers a more comprehensive and effective therapeutic regimen for numerous melasma pathologic causes. In the future, studies should investigate the best combination of various agents with microneedling and how to optimize therapeutic efficacy via optimal treatment routines.

#### 4.3.3 Microneedle Combined Therapy

Melasma treatment primarily eliminates melanosomes by facilitating their excretion via the inhibition of tyrosinase and associated proteins. Currently, a wide range of technologies, including Q‐switched lasers, picosecond lasers, and pulsed solid light are used for melasma treatment. However, the use of such technologies alone has limitations, such as the risk of severe responses and melasma recurrence. Therefore, for greater curative efficacy, we recommend combining various technologies targeted at distinct therapeutic targets. Multiple studies have demonstrated the superiority of combination therapy in melasma treatment and although previous studies have mainly focused on photoelectric therapy, there is growing interest in alternate therapeutic modalities (See Table 3 for details).

**TABLE 3:** Microneedle combined therapy.

Reference	Microneedle Type	Number of subjects	No of sessions (interval)	Treatment	Results	Side effects
Ustuner et al. [80]	Dermapen, 1.5 mm	15 females One male	12 (4 week)	Group I: QSNY + MN + AA; Group II: QSNY	QSNY+MN + AA is safe and effective in treating stubborn melasma	Transient erythema, HP, irritation
Quan et al. [81]	Dermaroller, 0.5 mm, 1 mm	90 females	4 (2 weeks)	Group I: QSNY + MN + compound repair of oligosaccharides Group II: Laser alone	QSNY + compound repair of oligosaccharides improves melasma better than QSNY only	Not mentioned
Jung et al. [82]	RF,1 mm	14 females One male	5 (2 weeks)	Right side: QSNY + RF; Left side: QSNY	QSNY+RF improves melasma better than QSNY only	Mild pain, discomfort tolerated by most patients
Kwon et al. [83]	FMR,0.5 ~ 1 mm	19 females Five males	10 (1 week)	Group I: QSNY + FMR; Group II: QSNY alone	QSNY + FRM improves melasma better than QSNY only	Group I had more mottled HP and rebound HP than Group II
Karadağ and Borlu [84]	Dermapen, 2 mm	15 females	5 (2 weeks)	QSNY + MN + biomimetic peptide formulation	QSNY+RF+ biomimetic peptides are safe and effective in treating melasma	Mild‐to‐moderate erythema
Lee et al. [86]	FMR,0.3 mm	25 females	5 (2 weeks)	Patients receive QSNY +RF on one side of the face, and QSNY only on the other side	QSNY+RF improved melanin index and half‐face MASI better than QCNY	Tolerable pain, erythema, and crusting
Debasmita et al. [85]	Dermaroller, 1.5 mm	42 females 18 males	5 (4 weeks)	Group I: Laser + 3% TA gel; Group II: MN + 3% TA gel	Both have the same efficacy in improving melasma	Burning sensation, pain, erythema more in Group II than Group I
Mekawy, Sadek and Seddeik Abdel‐Hameed [87]	Dermapen, 0.25 – 1 mm	30 females	6 (2 weeks)	Patients received MN + TA on one side of their face, and fractional ablative CO2 laser + 4 mg/mL TA on the other	Both MN and CO2 laser delivery of TA are safe and effective.	HP: 10% MN side
Mamdouh et al. [88]	Dermapen, 0.25 – 1 mm	30 females	4 (3 weeks)	Group I: Fractional CO2 laser + 5% TA cream; Group II: MN + 5% TA + 5% TA cream; Group III: 5% TA cream	TA+ MN or CO2 laser is superior to TA alone in improving melasma.	Recurrence: III > I > II; HP: I Dryness and transient irritation: II > I = III
Hofny et al. [89]	Dermapen, 2 mm	40 females	4 (4 weeks)	Group I:MN + 25% TCA; Group II: 25% TCA	MN + TCA is safe and effective in treating melasma	Burning sensation and enterythema: II > I; Folliculitis: I; HP: II

Abbreviations: FME, fractional microneedling radiofrequency; QSNY, QS‐Nd:YAG laser; TCA, trichloroacetic acid.

The combination of microneedle therapy with Qs‐Nd:YAG laser therapy is often used in melasma phototherapy studies [80, 81, 82, 83, 84, 85, 86, 87, 88]. For example, Ustuner et al. [80] treated 16 female patients with persistent melasma using vitamin C microneedle therapy combined with Qs‐Nd:YAG laser treatment and found that the combined treatment group exhibited significantly superior MASI score improvement when compared to the group treated with laser alone. This is probably because the Qs‐Nd:YAG laser generates heat, which promotes dermal blood circulation, thereby boosting microneedle mechanical activity and enhancing vitamin C transdermal absorption. Furthermore, Mekawy et al. [87] treated 30 female patients with melasma using six treatments of CO_2_ laser and microneedle therapy and observed that the control group (CO_2_ laser combined with 4 mg/mL tranexamic acid) and the combination group (microneedle therapy combined with 4 mg/mL of tranexamic acid) exhibited significantly reduced mMASI scores and that the difference between the two groups was not statistically significant, confirming that drug delivery using CO_2_ laser and microneedles is safe and efficacious.

Microneedle therapy has also exhibited melasma treatment potential when combined with other therapies, such as chemical stripping. In a trial that treated 40 female patients with melasma using 25% trichloroacetic acid and microneedling, Hofny et al. [89] observed that in the combined treatment group, the mMASI score was better when compared with the control group treated using 25% trichloroacetic acid alone. This is probably because trichloroacetic acid stripping removed excess melanin, promoting epidermal renewal, while microneedling enhanced trichloroacetic acid transdermal absorption and stimulated dermal hyperplasia. This study confirmed the safety and efficacy of 25% trichloroacetic acid when combined with microneedling.

Finally, combining microneedle therapy with other techniques offers a new melasma treatment method that can improve therapeutic efficacy while reducing adverse responses through the synergistic effects of several mechanisms. To improve treatment efficacy and patient satisfaction, future studies should investigate the optimal combination of microneedle therapy and other treatments and optimize treatment plans.

## 5 Conclusion

Microneedling, a skin therapy technique that creates controlled microinjuries, has gained popularity because it can cure various skin problems, including melasma. This comprehensive review examined the role of microneedling in melasma treatment, focusing on its therapeutic advantages and safety. Its advantages include a short recovery time, enhanced percutaneous topical medication absorption, and high tolerability across various skin types. Moreover, during melasma treatment, it exhibits few side effects, with the most prevalent one being transient redness and mild discomfort, which are handled well by patients [5].

Unlike traditional melasma therapies, microneedling preserves epidermal integrity promotes basement membrane regeneration, and does not significantly stimulate melanocyte proliferation. Additionally, it reduces the risk of hyperpigmentation, which, because of inflammation, is commonly associated with the healing process [90]. When combined with other treatment techniques, microneedle therapy has shown promise for reducing melasma incidence relapse and accompanying hyperpigmentation. However, because current clinical studies on melasma treatment using microneedling are hampered by small sample sizes, larger studies are needed to eliminate potential biases, including publication and follow‐up bias. Furthermore, microneedle combination therapies are limited, and investigating a broader range of combination protocols may be critical for improved therapeutic success. Future research efforts should also prioritize the determination of the optimal needle penetration depth and treatment regimen, which depend on the patient's skin tone. To maximize clinical outcomes for patients with melasma, emphasis should be placed on identifying the ideal needle length [91]. Taken together, current evidence highlights microneedling as a novel and effective melasma treatment option, with a good safety profile and few side effects. However, further research is needed to better understand its clinical applications and refine treatment regimens.

## Author Contributions

Wenwen Chen contributed to the writing of this paper, while Xingling Jian and Bo Yu were involved in its finalization.

## Conflicts of Interest

The authors declare no conflicts of interest.

## Data Availability

The data that support the findings of this study are available from the corresponding author upon reasonable request.
